# Use of data mining techniques to classify soil CO_2_ emission induced by crop management in sugarcane field

**DOI:** 10.1371/journal.pone.0193537

**Published:** 2018-03-07

**Authors:** Camila Viana Vieira Farhate, Zigomar Menezes de Souza, Stanley Robson de Medeiros Oliveira, Rose Luiza Moraes Tavares, João Luís Nunes Carvalho

**Affiliations:** 1 School of Agricultural Engineering, State University of Campinas, Campinas, São Paulo, Brazil; 2 Agricultural Informatics, Computational Intelligence Laboratory, Brazilian Agricultural Research Corporation (Embrapa), Campinas, São Paulo, Brazil; 3 Department of Soil, Rio Verde University, Rio Verde, Goiás, Brazil; 4 Brazilian Bioethanol Science and Technology Laboratory, Brazilian Center for Research in Energy and Materials, Campinas, São Paulo, Brazil; University of Vermont, UNITED STATES

## Abstract

Soil CO_2_ emissions are regarded as one of the largest flows of the global carbon cycle and small changes in their magnitude can have a large effect on the CO_2_ concentration in the atmosphere. Thus, a better understanding of this attribute would enable the identification of promoters and the development of strategies to mitigate the risks of climate change. Therefore, our study aimed at using data mining techniques to predict the soil CO_2_ emission induced by crop management in sugarcane areas in Brazil. To do so, we used different variable selection methods (correlation, chi-square, wrapper) and classification (Decision tree, Bayesian models, neural networks, support vector machine, bagging with logistic regression), and finally we tested the efficiency of different approaches through the Receiver Operating Characteristic (ROC) curve. The original dataset consisted of 19 variables (18 independent variables and one dependent (or response) variable). The association between cover crop and minimum tillage are effective strategies to promote the mitigation of soil CO_2_ emissions, in which the average CO_2_ emissions are 63 kg ha^-1^ day^-1^. The variables soil moisture, soil temperature (Ts), rainfall, pH, and organic carbon were most frequently selected for soil CO_2_ emission classification using different methods for attribute selection. According to the results of the ROC curve, the best approaches for soil CO_2_ emission classification were the following: (I)–the Multilayer Perceptron classifier with attribute selection through the wrapper method, that presented rate of false positive of 13,50%, true positive of 94,20% area under the curve (AUC) of 89,90% (II)–the Bagging classifier with logistic regression with attribute selection through the Chi-square method, that presented rate of false positive of 13,50%, true positive of 94,20% AUC of 89,90%. However, the (I) approach stands out in relation to (II) for its higher positive class accuracy (high CO_2_ emission) and lower computational cost.

## Introduction

Carbon dioxide (CO_2_) is continuously exchanged between soils and the atmosphere, mainly through the processes of photosynthesis and incorporation of organic matter derived from plants (efflux of CO_2_) and the decomposition of this organic matter by soil organisms (CO_2_ flux). Therefore, the amount of carbon (C) stored in the soils depends mainly on the equilibrium between the C inputs and outputs of the system [1].

In this context, increased C storage in soils worldwide could help compensate for the increase in anthropogenic CO_2_ emissions, while increased soil emissions could significantly worsen atmospheric rises [2].

Thus, the potential of agriculture to mitigate greenhouse gas emissions has been the subject of intense scientific research over recent years [1]. However, after many decades of research on soil organic matter decomposition, very few robust mathematical models and experiments to predict the effect of both biotic and abiotic factors on the soil C balance have been developed [3].

Considering this issue, the alternative that we propose is the use of data mining techniques to predict CO_2_ emission from the soil. Among the techniques of data mining, classification is a task that stands out in the studies of the scientific community of Knowledge Discovery in Database (KDD). The principle of this task is to predict a categorical variable, i.e., to discover a function that maps a set of records into a set of predefined variables, called classes. Such a function can be applied to new records in order to predict the class in which such records fit [4, 5].

Several algorithms can be used to perform such a task; however, some of them, such as J48, Naive Bayes, Multilayer Perceptron, SVM, Bagging, are widely used due to their usual good performance regarding classification processes. However, the models obtained through the use of different classifiers require assessment under the same conditions.

An alternative to compare predictive models is the Receiver Operating Characteristic (ROC) curve, which is a graphical method for evaluation, organization, and selection of diagnostic and/or prediction systems. This tool has been widely used in data mining processes to assess classification models and is particularly useful in areas presenting a great disparity between classes or when different costs/benefits must be taken into account for the different errors/correctness in classification [6].

Hence, our study aimed at using data mining techniques to predict the soil CO_2_ emission induced by crop management in sugarcane areas in Brazil. To accomplish that, we used different variable selection methods (correlation, chi-square, wrapper) and classification (Decision tree, Bayesian models, neural networks, support vector machine, bagging with logistic regression), and finally we compared the efficiency of different approaches through the ROC curve (Receiver Operating Characteristic).

## Materials and methods

### Site locations and experiment descriptions

The composition of the original database for this work was carried out by collecting soil variable (physical, chemical, microbiological) and climatic data at an experimental sugarcane area located in the city of Iracemápolis, São Paulo, Brazil (22°34’50” S, 47°31’07” W; 608 m above sea level), during the sugarcane crop reform period.

The soil under investigation is classified as Rhodic Hapludox, according to the Soil Taxonomy System [7], and is described as a Latosssolo Vermelho Eutroférrico, according to the Brazilian Soil Classification [8], with very clayey texture. In the site characterization, the soil presented a pH around 4.6, an average organic C content of 10.5 g C dm^–3^, base saturation of 50%, cation exchange capacity 10.25 cmol_c_ dm^–3^, bulk density of 1.34 kg dm ^-3^, from 0–40 cm depth. The distribution of particle sizes of sand, silt, and clay were 140, 194 and 666 g kg^–1^, respectively.

The data were obtained from a field experiment with a randomized complete block design in the split-plot design with four replicates. The plots involved treatments with and without cover crop with *Crotalaria Juncea* and subplots with two soil tillage conditions (minimum and conventional tillage). Each experimental unit (sub-plot) consisted of 15 lines of sugarcane, with a spacing of 1.5 m and 34 m in length.

### Analysis of CO_2_ emissions, soil temperature, and soil moisture

Soil CO_2_ emissions were recorded between April 27 and August 3, 2013. Emissions were measured in the morning (i.e. between 8 and 10 am) [9], using five PVC collars (diameter = 10 cm and height = 7 cm), inserted 0.02 m into the soil at each of the 16 plots (80 collars in total). The PVC collars were distributed in sugarcane field as follow: two of them located in the in-row, one in an intermediate position considered as seedbed region (at 0.38 m distance from in-row), and two in the center of the inter-row (at 0.75 m distance from in-row).

Soil CO_2_ emissions were measured using an infrared gas analyzer (IRGA) produced by LI-COR (model LI8100A, Nebraska, USA). This chamber was a closed system with an internal volume of 991 cm^3^ and a contact area with the soil of 71.6 cm^2^. When recording emissions, the chamber was placed over the PVC collars to prevent any mechanical disturbance to the soil profile, which may result in overestimation of emissions.

The quantification of CO_2_ emissions began 24 hours after soil tillage operations. In each plot, the CO_2_ emission measurement lasted 90 seconds. The measurements were taken for 97 days days after soil tillage, until CO_2_ emissions had stabilized. The CO_2_ emissions recorded from the five collars within each plot (two in intra-rows and three in inter-rows) were aggregated into a single measure using a weighted average (assuming an area of 27% for the rows and 73% for the inter-rows). Soil CO_2_ emission over the entire study period was estimated by the integral of the area formed below the emission curves over time.

The measurements of soil temperature and soil moisture were taken at exactly the same time as soil CO_2_ measurements. Soil temperature (St) was assessed at all points studied with the sensor which is part ofthe same LI-COR instrument (model LI8100A). This sensor consists of a 20 cm rod inserted into the soil perpendicular to the surface near the PVC collars used to measure the soil respiration. Soil moisture (Sm) was measured simultaneously with the measurement of CO_2_ concentration using Time Domain Reflectrometry (TDR). Probe Thetaprobe ML2 (Delta-T Devices, Cambridge, UK) is an English manufacturing tool that directly measures the water content in the soil, corresponding to the volumetric moisture content, using the principle of wave generation which releases an electromagnetic pulse to a set of rods with reflection measured in TDR. Moreover, during experimental period, rainfall events were monitored through an automatic surface weather station installed at the experiment site.

In the end of the CO_2_ sampling period, disturbed and undisturbed soil samples were collected at the depths of 0.00–0.10 m, 0.10–0.20 m, 0.20–0.30 m, and 0.30–0.40 m.to evaluate the effects of the treatments on soil attributes.

### Soil physical, chemical and biological variables

Total soil porosity (Sp), macroporosity (Macro), microporosity (Micro), and bulk density (Bd) were analyzed according to the Brazilian Agricultural Research Corporation methodologies [10]. The soil resistance to penetration (RP) was obtained using the Stolf´s formulation [11]. The mean diameter of the aggregates (MDA) was determined according to the method described by Kemper and Chepil [12] and the calculation of the aggregate tensile strength (Ts) was performed as described by Dexter and Kroesbergen [13].

All samples were taken to the laboratory, air dried and subsequently passed through a 2.0 mm mesh. We measured soil pH (CaCl_2_ 0,01 mol L^-1^), exchangeable cations (Ca^2+^, Mg^2+^ and K^+^), phosphorus available in resin (P), organic C concentration (wet oxidation), acidity potential, CEC potential, and base saturation in accordance with the methodology proposed by Raij [14].

For the analysis of microbial biomass C, the samples were collected and placed in a cooler and refrigerated during the transportation to the laboratory for preservation in cold storage at 4°C until analysis. The microbial biomass C (MBC) was determined through the fumigation-extraction method proposed by Vance [15].

### Data mining

The original dataset was constituted of 19 variables (one dependent or response variable and 18 independent or explanatory variables) (Table 1) which were added to the dataset numbering 1,552 observations. The variable-target refers to the soil CO_2_ emission and is the classification target.

**Table 1:** Description of the 19 variables (independent and dependent) used in the database composition.

Variable	Type	Abbreviation	Description	Unit
**Physical**	Independent	Sm	Soil moisture	%
	Independent	St	Soil temperature	°C
	Independent	MDA	Mean diameter of the aggregate	mm
	Independent	Macro	Soil macroporosity	%
	Independent	Micro	Soil microporosity	%
	Independent	TP	Total porosity	%
	Independent	Bd	Bulk density	kg dm ^-3^
	Independent	PR	Penetration resistance	MPa
	Independent	Ts	Tensile strength of the aggregate	kPa
**Chemical**	Independent	H+Al	Acidity potential	cmol_c_ dm ^-3^
	Independent	Al^3+^	Exchangeable aluminum	cmol_c_ dm ^-3^
	Independent	Ca^2+^	Exchangeable calcium	cmol_c_ dm ^-3^
	Independent	Mg^2+^	Exchangeable magnesium	cmol_c_ dm ^-3^
	Independent	K^+^	Exchangeable potassium	cmol_c_ dm ^-3^
	Independent	P	Exchangeable phosphorus	mg dm ^-3^
	Independent	Organic C	Soil Organic Carbon	mg dm ^-3^
**Microbiological**	Independent	MBC	Microbial biomass carbon	Ug C g^-1^
**Climatic**	Independent	P	Rainfall	mm day ^-1^
**Variable-target**	Dependent	CO_2_ emission	soil CO_2_ emission	kg ha^-1^ dia^-1^

The 18 independent variables were constituted of nine soil physical attributes (Sm, Ts, MDA, Macro, Micro, TP, Bd, PR, Ts), seven soil chemical attributes (H+Al, Al^3+^, Ca^2+^, Mg^2+^, K^+^, P, C), one soil microbial attribute (MBC), and a climatic variable (daily rainfall obtained at a weather station located in the sugarcane mill) (Table 1).

The data were initially evaluated by through descriptive statistics through Box-plot graphs. The box shows the data between the first and third quartiles (hinge), with median represented by a line inside the box. Vertical lines (whiskers), starting in the middle of the base (and top) of the box and ending in values (referred to as adjacent lower and upper) approximately indicate the variability of the data [16].

In order to identify different levels of CO_2_ emissions from the soil, a goal attribute discretization was required. For this purpose, the CO_2_ emission values (kg ha^-1^ dia^-1^) were organized in ascending order and divided equally into three emission classes: low, medium and high (Table 2).

**Table 2:** Distribution of CO_2_ emission (kg ha^-1^ dia^-1^) according to the low, medium and high classes and their limits.

Class	Limit
Low	[3.8; 29.95]
Medium	[29.96; 53.63]
High	[53.64; 770.5]

**Fig 1:**
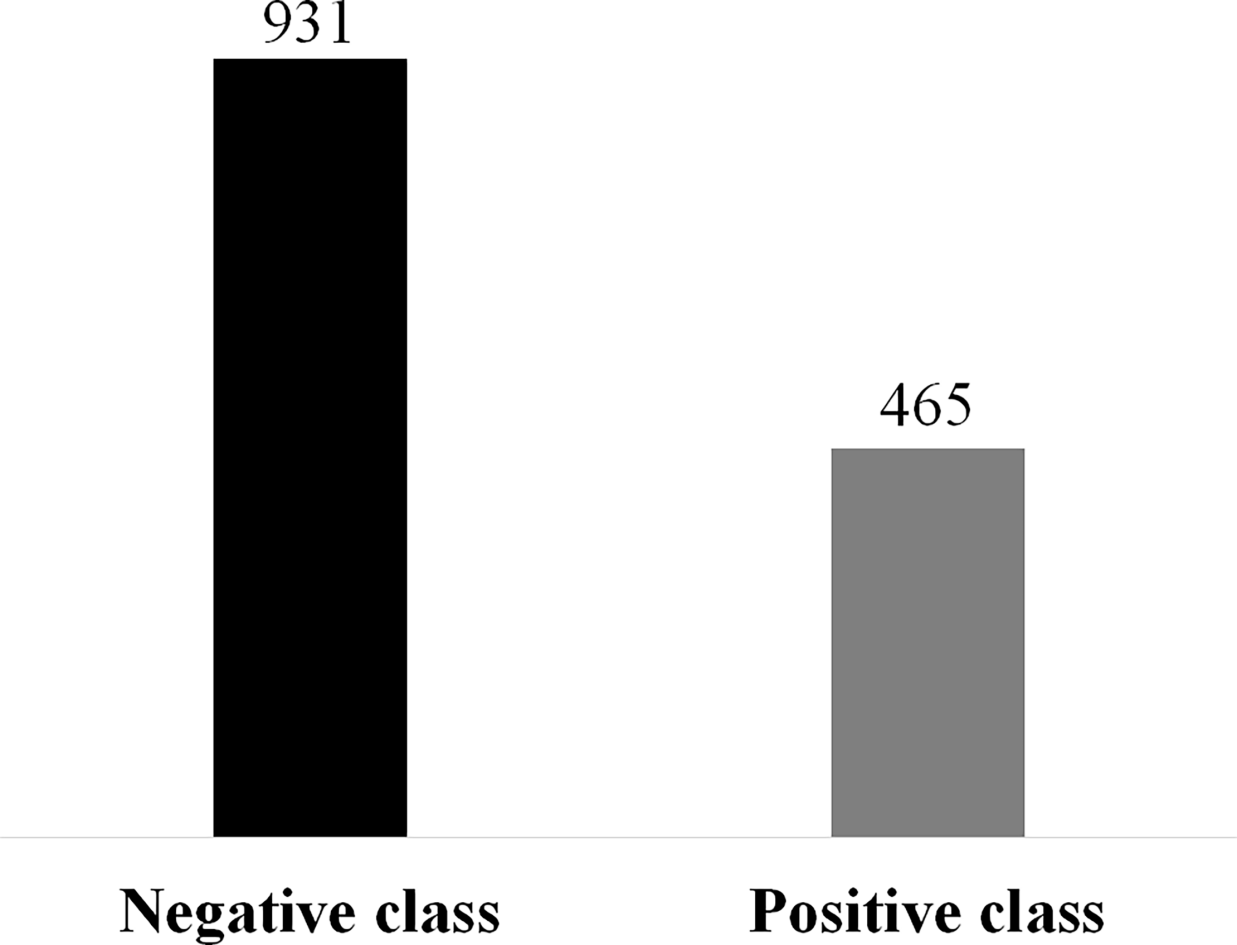
Discretization of CO_2_ emission from the soil in the positive class (CO_2_ emissions from high soil) and the negative classes (medium and low CO_2_ emissions).

In order to compare the performance of different classifiers through the ROC curve, a new discretization was performed considering the high class belonging to the positive class and the remaining classes (low and medium) were grouped to form the negative class (Fig 1**)**.

**Fig 2:**
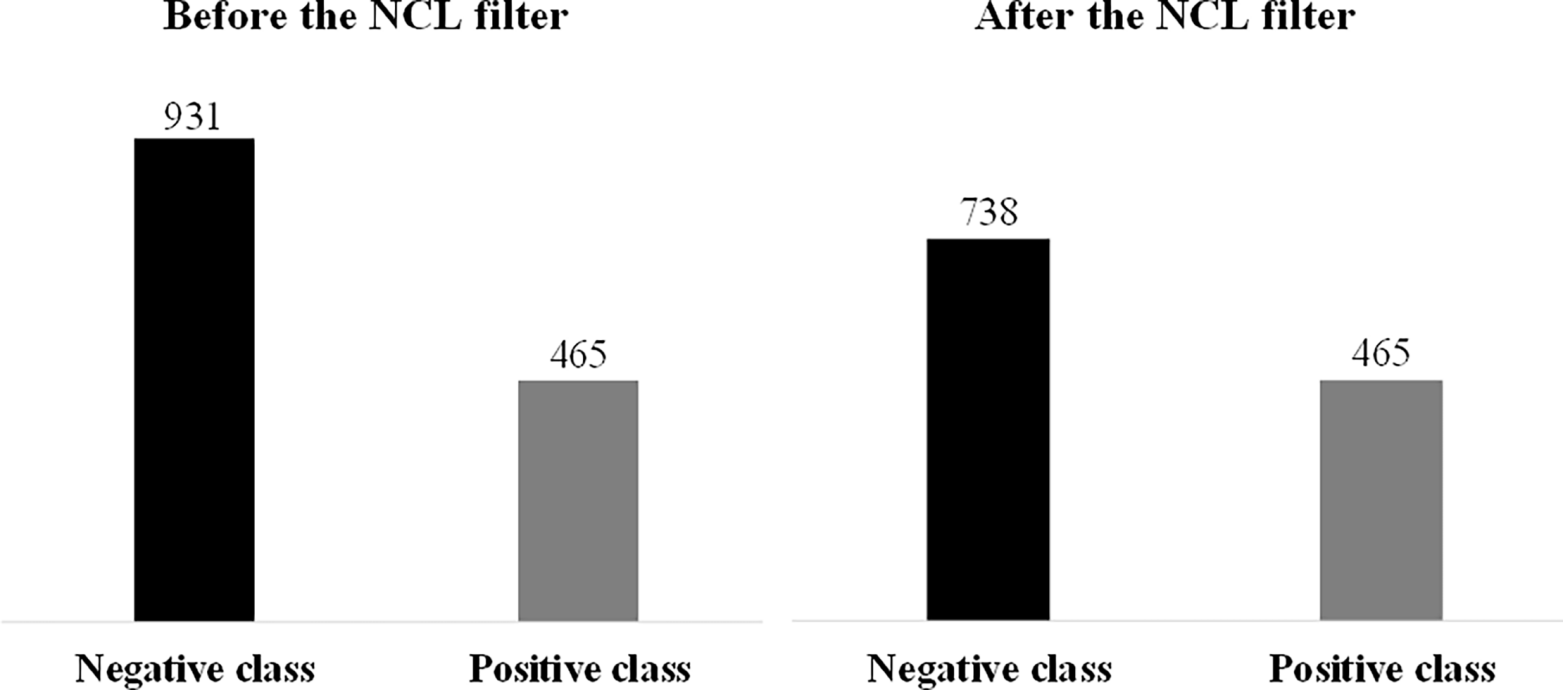
Demonstration of the classes distribution before and after the application of the NCL filter.

To do so, an imbalance between the classes occurred since the negative class obtained 2/3 of the data and the positive class kept only 1/3, hampering the classifiers to learn the positive class. For the purpose of improving the accuracy of the model and mitigating the problem of unbalanced classes, the "under sample" method was applied to balance the number of observations per class. This method is used in the training process to slightly reduce the observations of the major (negative) class and allow the (positive) minor class to be learned by the classifier, considering that the interest class is most often the positive class. For this purpose, the Stratified Remove Folds Filter was used to select two subsets, one for training, containing 90% of the original dataset, and one for testing, with 10% of the original dataset. Subsequently, we applied the Neighborhood Cleaning Rule (NCL) filter) available in Weka software 3.4.13. The distribution of classes in the database after this operation is shown in Fig 2.

From this subset, the selection of variables was performed using different methods:

1. Absence of attribute selection upon the occurrence of use of all data.
2. Correlation-based feature selection (CFS) searching for the set of correlated variables to prevent the reuse of the same information [17].
3. The chi-square method (χ^2^) is based on the concept of statistical independence.
4. Wrapper method, which functions as a "black box". In order to find the subset of variables that most satisfactorily fits the classification algorithm [18].

From the variables selected through each selection method, different classification algorithms were tested:

1. J48—Decision tree [19].
2. Naïve Bayes—Bayesian Classifier [20].
3. Multilayer Perceptron—Neural networks.
4. SMO (Sequential Minimal Optimization)—Vector Machine Support [21].
5. Bagging with Logistic Regression—Meta classifier [22].

The different approaches were validated using the supplied test set method (90% of the database for training and 10% for testing) and two metrics: (i) accuracy rate; (ii) Kappa coefficient.

The induction of the decision tree model resulted in the calculation of the known matrix of errors, or matrix of confusion (Table 3), widely used in statistical analysis of agreement [23].

**Table 3:** A 2x2 matrix of confusion. TP = true positive; FP = false positive; FN = false negative; TN = true negative.

		PREDICT		
		Class A	Class B	Total
**TRUE**	**Class A**	TP = (A, A)	FN **=** (A, B)	P
	**Class B**	FP **=** (B, A)	TN = (B, B)	N
**Total**		P’	N’	P+N

Column ‘Total’ in Table 3 presents P as the total value of positive cases and N as the total of existing negative cases in the training set. In the Total, P' is the total number of cases that the model rated as positive cases and N' the total number of cases classified as negative. From the matrix of confusion, it is possible to extract the performance evaluation metrics. The rate of accuracy is the percentage of examples that were correctly classified by the classifier and can be expressed according to Eq 1.

Accuracy=(VP+VN)/(P+N) (1)

To describe the measure of agreement between the predicted and observed classes, which deducts the expected number of correct answers (using a classification at random) of the actual number of the accuracy of the classifier, we used the Kappa measure (Eq 2). Kappa values vary from 0 to 1, representing bad and good classification results, respectively. The Kappa coefficient can be defined by the following equation [23]:

K=Pr(a)−Pr(e)/1−Pr(e) (2)

where Pr (a) is the relative agreement observed for a given class in the matrix of confusion; Pr (e) is the probability of expected agreement for this same class.

The Kappa coefficient is calculated taking into account all classes. A possible interpretation of the performance of the models from this measure was introduced by Landis and Koch [24].

In order to compare the performance of the different classifiers associated with the attribute selection methods, the ROC curve was used, a graphical tool used to assess the algorithm using software Roc on 2.0.

The analysis of the curves generated by each classification model in the ROC space is represented by the ratio between the true positive rate (TPR) axis and the false positive rate (FPR). The classifiers positioned at the bottom of the "convex hull" have a precision rate lower than those contained in it. In addition, the larger the area under the curve, the greater the accuracy of the model. The point (0, 0) represents the strategy of never classifying an example as positive. Models that correspond to this point do not present any false positive, but are unable to classify any true positive either. The inverse strategy, of always classifying a new example as positive, is represented by the point (100%, 100%). The dot (0, 100%) represents the perfect model, i.e., all positive and negative examples are correctly classified. In contrast, the point (100%, 0) represents the model that always makes erroneous predictions [6].

## Results

**Fig 3:**
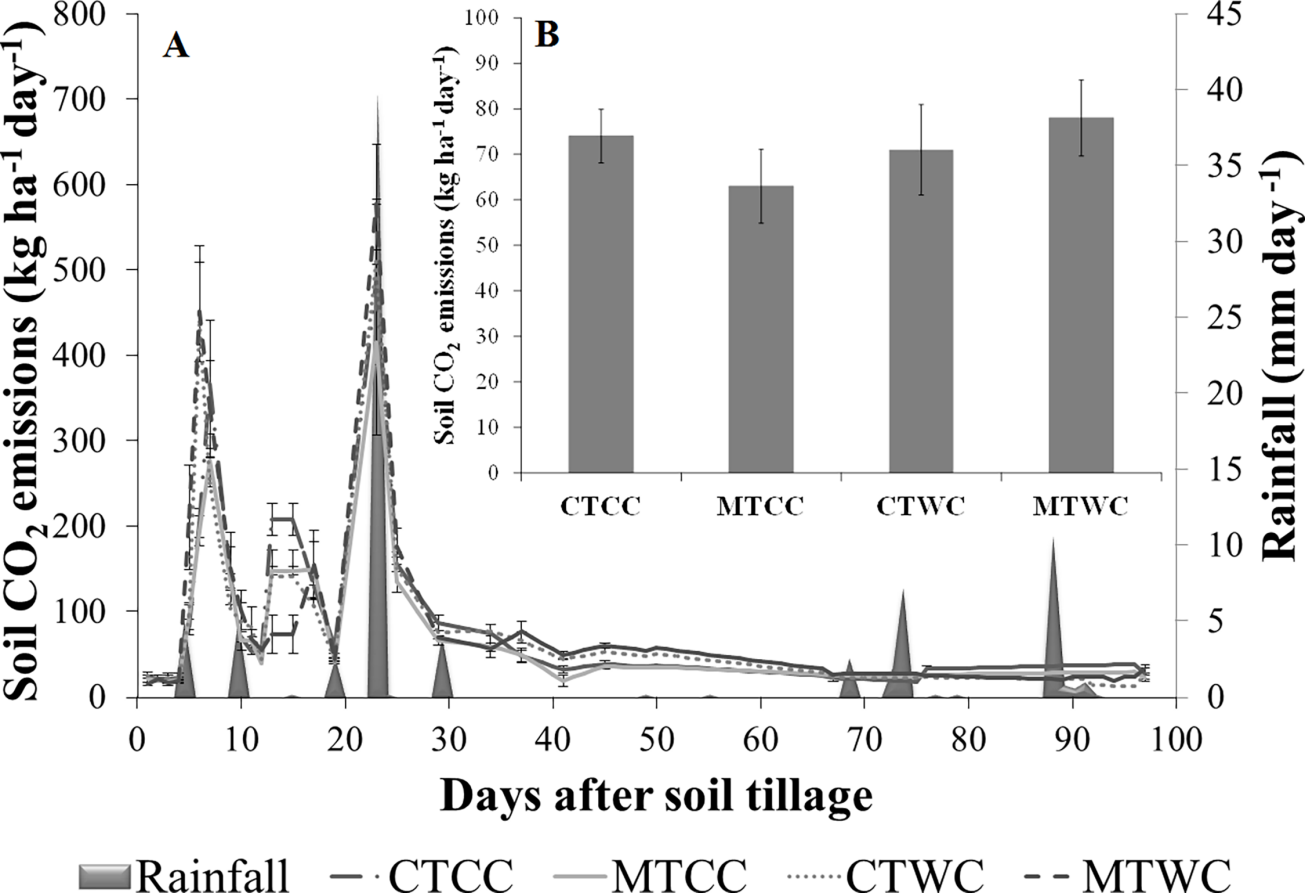
**(A) Temporal variability of soil CO**_**2**_ **emission and rainfall, and (B) average soil CO2 emission with their respective standard deviations, in the different management systems used in the experimental area.** CTCC = Conventional tillage with cover crop, MTCC = Minimal tillage with cover crop, CTWC = Conventional tillage without cover crop, MTWC = Minimal tillage without cover crop.

The behavior of soil CO_2_ emission for each treatment along the experimental period is presented in Fig 3. It was possible to observe great variability of the CO_2_ emission, mainly in the first days after the soil tillage practices and after rainfalls events, reaching emission values of up to 771 kg ha^-1^day^-1^ (Fig 3A). Around the 40^th^ day after tillage, there was a tendency of stabilization of soil CO_2_ emissions, independent of rainfall events in the experimental area.

Analyzing the average soil CO_2_ emissions, it was observed that the treatment where conservationist practices were used (cover crop and minimal tillage—MTCC) lower median values of CO_2_ emission was observed compared to other treatments (Fig 3B), which presented average CO_2_ emission of 63 kg ha^-1^ day^-1^ versus 74 kg ha^-1^ day^-1^ of the treatment CTCC, 71 kg ha^-1^ day^-1^ of the standard treatment CTWC, and finally 78 kg ha^-1^ day^-1^ of the MTWC.

**Fig 4:**
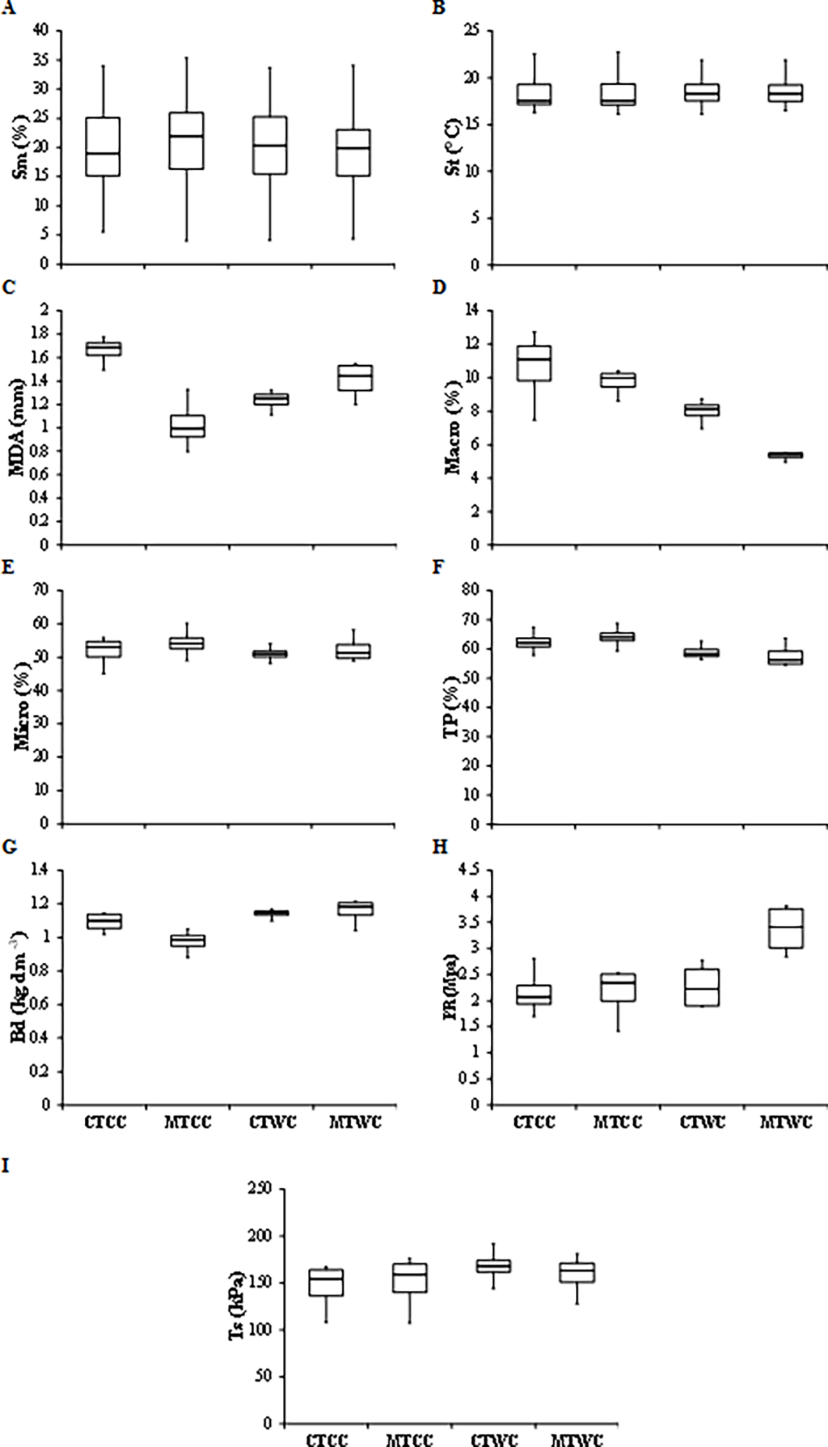
Box plot for the physical variables of the soil in the different management systems used in the experimental area. CTCC = Conventional tillage with cover crop, MTCC = Minimal tillage with cover crop, CTWC = Conventional tillage without cover crop, MTWC = Minimal tillage without cover crop. Sm = Soil moisture, St = Soil temperature, MDA = Mean diameter of the aggregate, Macro = Soil macroporosity, Micro = Soil microporosity, TP = Total porosity, Bd = Bulk density, PR = Penetration resistance, Ts = Tensile strength of the aggregate.

Soil moisture was another attribute that presented high variability along the evaluated period. Soil moisture was influenced by rainfall events, where its values ranged from 4% to 36%. The soil temperature presented small variability among the treatments. In general, a tendency of small average temperature was observed in cover crop treatments (Fig 4).

**Fig 5:**
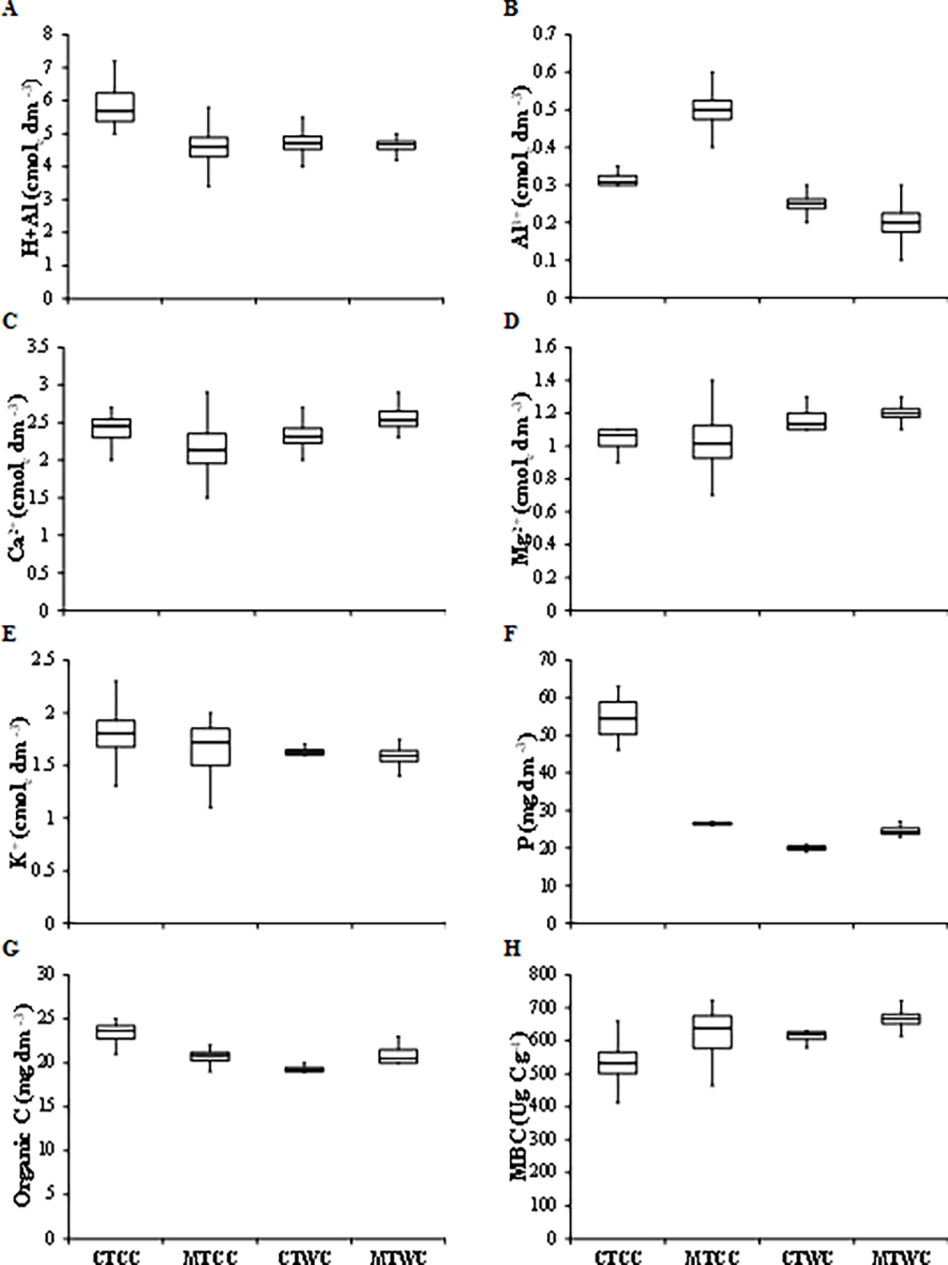
Box plot for soil chemical and biological variables in the different management systems used in the experimental area. CTCC = Conventional tillage with cover crop, MTCC = Minimal tillage with cover crop, CTWC = Conventional tillage without cover crop, MTWC = Minimal tillage without cover crop, H+Al = Acidity potential, Al^+3^ = Exchangeable aluminum, Ca^+2^ = Exchangeable calcium, Mg^+2^ = Exchangeable magnesium, K^+^ = Exchangeable potassium, P = Exchangeable phosphorus, Organic C = Organic Carbon, MBC = Microbial biomass carbon.

In addition, it can be observed that the areas under cover crop cultivation presented higher porosity (macro, micro e TP), Al^+3^, K, P, Organic C e and lower Bd, PR, RT, Mg+^2^. Specifically for the MTCC treatment, it was observed that in this treatment occurred greater Sm, Micro, TP, Al^+ 3^, K^+^, MBC associated with lower MDA, Bd, H + Al^+ 3^, Ca^+ 2^, Mg^+ 2^ (Figs 4 and 5).

According to the results presented in Table 4, the number of variables selected differed among the assessment methods, which has occurred since the selection of only one attribute, as in the case of the CFS method, having selected soil temperature exclusively, until the selection of ten variables, as in the case of method χ^2^ (Table 4).

**Table 4:** Variables selected through the different selection methods.

Methods	Selected variables	Total
χ^2^	Sm, St, rainfall, Macro, Tp, Bd, PR, pH, Al, P	10
CFS	St	1
Wrapper J48	Sm, St, rainfall, Macro, Bd, Al, Ca, Organic C	8
Wrapper Naïve Bayes	St, rainfall, Macro, pH	4
Wrapper SMO	St, rainfall, MDA, Tp, Organic C	5
Wrapper Mult. Perceptron	St, Sm, pH, H+Al, P, Organic C	6
Wrapper Bagging Logistic	Sm, St, rainfall, MBC, Ts, pH, Mg, Organic C	8

When analyzing the variables selected in the different approaches, we observed the recurrence of some of them, such as: soil temperature, rainfall, soil moisture, pH, and soil organic C (Table 4). Soil temperature appears in all attribute selection methods, followed by rainfall, present in six out of the seven approaches assessed, and the three variables, soil moisture, pH, and soil organic C, which appear in four of the seven methods tested.

Among the algorithms assessed for soil CO_2_ emission classification, J48, Naïve Bayes and Bagging with logistic regression presented better performance when using the χ^2^ method for attribute selection, with the accuracy rate of the different approaches ranging from 87.18 to 84.61 and Kappa coefficient from 0.73 to 0.66. In contrast, the SMO and Multilayer Perceptron algorithms presented a subset of variable selected through the Wrapper method with an accuracy rate varying from 89.10 to 85.90 and Kappa coefficient from 0.77 to 0.69 (Table 5).

**Table 5:** The performance of different classifiers associated with the attribute selection methods assessed.

Approaches	J48		Naive Bayes		SMO		Multilayer Perceptron		Bagging Logistic	
	Accuracy	Kappa	Accuracy	Kappa	Accuracy	Kappa	Accuracy	Kappa	Accuracy	Kappa
**Without attribute selection**	87,18	0,72	84,62	0,66	83,97	0,65	86,54	0,72	84,62	0,67
**χ^2^**	**87,18**	**0,73**	**84,61**	**0,66**	84,63	0,66	87,18	0,73	**84,62**	**0,68**
**CFS**	84,62	0,68	82,69	0,64	83,97	0,67	83,97	0,67	82,69	0,65
**Wrapper**	85,90	0,70	82,05	0,60	**85,90**	**0,69**	**89,10**	**0,77**	83,97	0,67

The IV approach provided higher AUC and TP, being 89.90 and 94.2, respectively, followed by the V approach, which presented an AUC of 89.85 and a TP of 94.2. In contrast, the III approach provided the lowest AUC, 85.10 and TP, 82.7, against the remaining approaches (Table 6).

**Table 6:** Rate of false positive (FP), true positive (TP) area under the curve (AUC) for the best associations between classification and attribute selection method.

Approaches		FP Rate	TP Rate	AUC
**I**	J48 **x** χ^2^	15,4	92,3	89,44
**II**	Naive Bayes **x** χ^2^	12,5	78,8	89,16
**III**	SMO **x** Wrapper	12,5	82,7	85,10
**IV**	Multilayer Perceptron **x** Wrapper	13,5	94,2	89,90
**V**	Bagging Logistic **x** χ^2^	20,2	94,2	89,85

FP Rate = %; TP Rate = %; AUC = %.

**Fig 6:**
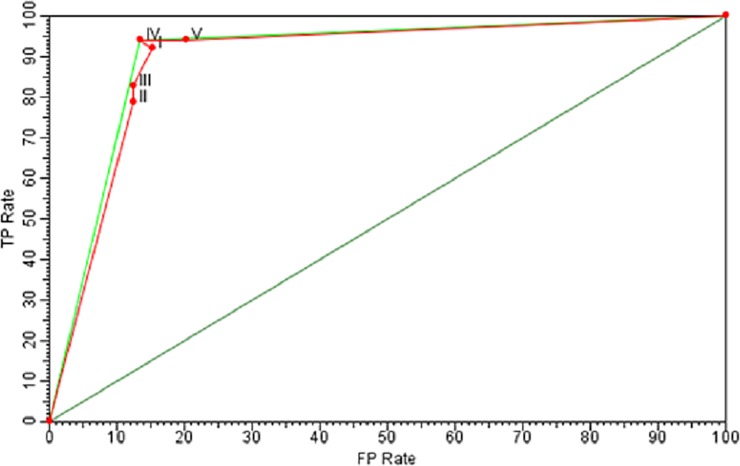
Performance in the ROC (Receiver Operating Characteristic) space of the different approaches used to classify soil CO_2_ emission, considering the high CO_2_ emission class as the "most important" class. I = J48 with χ^2^, II = Naïve Bayes with χ^2^, III = Bagging with logistic regression with χ^2^, IV = SMO using wrapper method, V = Multilayer Perceptron with wrapper method.

Regarding the analysis of the different approaches through the ROC curve, in general, all of them had a good performance regarding the classification, since none of them remained below the random performance line (Fig 6). In addition, we observed that the best approaches were IV and V for being the only ones to be encompassed by the convex hull. However, the IV approach has advantages over the V for being closer to the point (0.0 and 100).

## Discussion

### Effect of the cover plant and soil tillage to mitigate CO_2_ emissions

In spite of the high variability of CO_2_ emission, the extreme values were not considered as outliers, since they were associated by tillage operations and rainfall events in the experimental area.

The magnitude of CO_2_ loss from soil influenced by tillage practices is highly related to the intensity of soil disturbance caused by tillage which alters soil organic matter decomposition environment due soil aeration, break the aggregates, incorporation of residue into the plow layer [25, 26].

Studies conducted under Brazilian conditions confirm this tends, i.e., Iamaguti [27], evaluated the CO_2_ emissions influenced by three systems of soil tillage in sugarcane replanting area, observed that for the conventional tillage the exposed and disaggregated soil favors higher losses of CO_2_. In a study on quantification the impact of sugarcane, harvest systems, straw and soil management on soil CO_2_ emissions, Figueiredo [28], showed that intensive soil tillage and the incorporation of sugarcane straw into the soil increased short-term CO_2_ emissions.

Rainfall events, in turn, promoted the increase of soil moisture and stimulated CO_2_ emissions, producing CO_2_ emissions belonging to the high class. This fact is supported by several studies in sugarcane fields that demonstrate the influence of rainfalls events on soil CO_2_ emissions due to changes in soil water content [28] [29] [30].

The soil moisture is an attribute essential for soil microbial metabolism, and can present both direct and indirect influence under CO_2_ emissions. Adequate soil moisture tends to stimulate microbial activity in the soil rather than inhibit. According to Vicent [31] optimum soil moisture values range from 25% to 40%, above this range the CO_2_ emission is limited by excess water and lack of oxygen in the soil, and below limits soil respiration by drought. In our study, the average value of soil moisture remained around 20%, whose maximum soil moisture value was 36%, not exceeded, therefore, values that could compromise the availability of O_2_ in the pore space of the soil.

However, after the 40^th^ day after soil tillage we observed a tendency to stabilize CO_2_ emissions. The same occurred in other studies performed by Panosso [32] and La Scala [33]. This is probably associated to decrease of the labile C caused by tillage [34, 35].

Our results also showed that the association between the cover crop and minimum tillage were efficient methods to promote the mitigation of soil CO_2_ emissions in sugarcane fields. These results are in agreement with Figueiredo [28], Tanveer [36] and Moitinho [37], that observed a lower CO_2_ emission due to the reduction of the frequency of tillage and maintenance of crop residues on soil surface.

The identification of the factors influencing the emission of CO_2_ from the soil in agricultural areas opens up opportunities for adopting practices that reduce the net emissions of this gas [27]. In a study on the effect of different soil tillage systems on CO_2_ emissions, Lu [38], observed that the average loss of C in the form of CO_2_ was significantly lower in the treatment with less soil disturbance (no-tillage) compared to conventional tillage. The authors pointed out that the lower soil temperature presented by no-tillage treatment reduces microbial activity and, consequently the emission of CO_2_ in the soil. Another explanation for the lower CO_2_ emissions in no-tillage treatment could be greater physical protection of the C inside the soil aggregates.

Moreover, the maintenance of crop residues on the soil surface in areas where soil disturbances are minimal, limits soil-residue contact, resulting in a reduced rate of decomposition, and consequently lower CO_2_ emissions in these systems [25, 39].

The adoption of cover crop and minimum tillage (CTCC) provided desirable modifications in soil attributes, such as: greater Sm, Micro, TP, K^+^, MBC, and lower, Bd. In agreement, Torres [40], evaluated changes in the soil physical attributes with the use of different cover crops under no-tillage system, and observed positive changes in physical attributes in the soil surface layer. Similar results were also found by Lu [38], who observed significant changes in soil properties through the conversion of the conventional tillage system to no-tillage. However, it is important to highlight that our results represent a case study with one soil type and with specific climatic conditions, and more studies should be performed in order to analyze the results in different edaphoclimatic conditions.

### Classification of CO_2_ emissions

The variables of soil moisture, soil temperature, rainfall, pH, and soil organic C showed high predictive power regarding the classification of soil CO_2_ emission and were frequently selected through the different methods of attribute selection assessed in this study. Several studies confirm the direct and/or indirect influence of these variables on the soil CO_2_ emission.

For example, La Scala Junior [33], Corradi [41], Teixeira [42] found direct correlations between soil moisture and CO_2_ emissions under different conditions. Iamaguti [27], Karhu [43] and Tavares [44] observed a significant correlation between soil temperature and soil CO_2_ emission. Moitinho [29] and Silva-Olaya [30] observed higher CO_2_ emissions along days with rainfall events. Fuentes [45] and Marcelo [46] reported that increasing soil pH affects the activity and microbial population in the soil and consequently their CO_2_ emission. Reuss and Johnson [47] and Tossell [48], point out that carbonic acid, produced by the dissolution of CO_2_ in water, is an important acidifying agent in natural systems. La Scala Junior [49] observed a linear correlation between CO_2_ emission and Organic C.

According to Park [50], the closer the AUC is to the value 1, the better the overall performance of the test, which makes a test with AUC = 1 be regarded as perfectly accurate; therefore, the higher the AUC, the better the overall performance of the approach used. In this sense, the IV approach presented higher AUC, being superior to all of the remaining approaches assessed; however, the difference between IV and V, I and II approaches was very low, within the order of 0.05, 0.46 and 0.74, respectively.

In contrast, the analysis of the different approaches in the ROC space proved both the IV and the V as the most satisfactory for having been the only approaches to be encompassed by the convex hull. However, approach IV tends to have advantages over V since it is closer to the point (0.0 and 100%). Prati [6] point out that an optimal model should be as close as possible to the point (0 and 100%). In addition, Fawcett [51] refers to the ROC chart as a description of the relative compensation between the benefits (true positive) and costs (false positives) of a classification. Thus, a point located in the upper left corner demonstrates that a higher amount of positive and negative examples are classified correctly, consequently generating a lower classification cost, corroborating with the statement that the IV approach is the best one.

Through their research, several authors have found good performance both in the attribute selection Wrapper method and in the Multilayer Perceptron classifier. Liu and Yu [52] point out to a variety of available attribute selection algorithms; however, the attribute selection Wrapper method has generally performed better than other methods for selecting a subset of variables that are more suitable to the predetermined mining algorithm, but also tending to demand high computational effort and cost, especially in datasets with great dimensionality, which makes it possibly not suitable to some mining algorithms. In agreement with the results, Hall and Geoffrey [53], by comparing several methods of variables selection for the supervised classification, also concluded that the Wrapper method was the most satisfactory for the selection.

Finally, Freitas [54] investigated the use of the classifier Multilayer Perceptron for the predicting of the spatial variability of soil CO_2_ emissions in sugarcane areas, also concluding that this approach satisfactorily met the expectations having proved good application potential for this type of issue.

## Conclusions

The CO_2_ emissions present high variability, mainly in the first days after the soil tillage, in which soil disturbance caused by tillage and rainfall events stimulate CO_2_ production, while the association between cover crop and minimum tillage are effective strategies to promote the mitigation of soil CO_2_ emissions.

Data mining techniques to predict soil CO_2_ emission proved promising results for having allowed the development of several classifications approaches with high accuracy rate (above 80%), with variables such as soil moisture, soil temperature, rainfall, pH, and organic C showing high predictive power. Finally, among the approaches assessed in this study, the Multilayer Perceptron approach (a particular case of artificial neural networks) with the Wrapper attribute selection method tends to be more advantageous for its higher accuracy of the positive class with a consequent lower cost of classification.

Finally, we highlighted that the data presented herein represent a short-term case study conducted in specific edaphoclimatic conditions. Comprehensive studies evaluating the efficiency of the data mining techniques to predict the soil CO_2_ emissions should be encouraged to test the reproducibility of these techniques under different soil and climate conditions.

## Supporting information

S1 Table Daily CO_2_ emissions. Day = Days after soil tillage and replanting of sugarcane; Factor 1 = Cover crop; Factor 2 = Soil tillage; Block = Experimental design in randomized blocks; CO_2_ = Soil CO_2_ emissions.
(XLSX)

S2 Table Soil physical attributes. Factor 1 = Cover crop; Factor 2 = Soil tillage; Block = Experimental design in randomized blocks; Sm = Soil moisture; St = Soil temperature; MDA = Mean diameter of the aggregate; Macro = Soil macroporosity.; Micro = Soil microporosity; TP = Total porosity; Bd = Bulk density; PR = Penetration resistance; Ts = Tensile strength of the aggregate.
(XLSX)

S3 Table Soil chemical attributes. Factor 1 = Cover crop; Factor 2 = Soil tillage; Block = Experimental design in randomized blocks; H+Al = Acidity potential.; Al^+3^ = Exchangeable aluminum; Ca^+2^ = Exchangeable calcium; Mg^+2^ = Exchangeable magnesium; K^+^ = Exchangeable potassium; P = Exchangeable phosphorus; Organic C = Organic Carbon.
(XLSX)

S4 Table Soil biological attributes. Factor 1 = Cover crop; Factor 2 = Soil tillage; Block = Experimental design in randomized blocks; MBC = Microbial biomass carbon.
(XLSX)
